# Active and latent tuberculosis in Brazilian correctional facilities: a cross-sectional study

**DOI:** 10.1186/s12879-015-0764-8

**Published:** 2015-01-22

**Authors:** Andrea da Silva Santos Carbone, Dayse Sanchez Guimarães Paião, Renata Viebrantz Enne Sgarbi, Everton Ferreira Lemos, Renato Fernando Cazanti, Marcos Massaki Ota, Alexandre Laranjeira Junior, José Victor Bortolotto Bampi, Vanessa Perreira Fayad Elias, Simone Simionatto, Ana Rita Coimbra Motta-Castro, Maurício Antonio Pompílio, Sandra Maria do Valle de Oliveira, Albert I Ko, Jason R Andrews, Julio Croda

**Affiliations:** University Hospital, Federal University of Grande Dourados, Dourados, Brazil; Faculty of Health Sciences, Federal University of Grande Dourados, Dourados, Brazil; Faculty of Ambiental and Biological Sciences, Federal University of Grande Dourados, Dourados, Brazil; Department of Biochemical Pharmacy, Federal University of Mato Grosso do Sul, Campo Grande, MS Brazil; Oswaldo Cruz Foundation, Campo Grande, Brazil; Faculty of Medicine, Federal University of Mato Grosso do Sul, Campo Grande, MS Brazil; University Hospital, Federal University of Mato Grosso do Sul, Campo Grande, Brazil; Gonçalo Moniz Institute, Oswaldo Cruz Foundation, Salvador, Brazil; Department of Epidemiology of Microbial Disease, Yale School of Public Health, New Haven, CT USA; Division of Infectious Diseases and Geographic Medicine, Stanford University School of Medicine, Stanford, CA USA

**Keywords:** Tuberculosis, Prisoners, TST, Infection, Active case detection, Screening cross-sectional study, Brazil, Epidemiology

## Abstract

**Background:**

Tuberculosis (TB) rates among prisoners are more than 20 times that of the general population in Brazil, yet there are limited data available to facilitate the development of effective interventions in this high-transmission setting. We aimed to assess risk factors for TB infection and evaluate the yield of mass screening for active disease among inmates.

**Methods:**

We administered a questionnaire and tuberculin skin test (TST) to a population-based sample of inmates from 12 prisons in Central-West Brazil and collected sera for HIV testing and two sputum samples for smear microscopy and culture from participants reporting a cough of any duration. Hierarchical Poisson regression models were used to evaluate factors associated with latent tuberculosis infection (LTBI).

**Results:**

We recruited 3,380 inmates, of which 2,861 (84.6%) were males from 8 prisons, and 519 (15.4%) were females from 4 prisons. Among the 1,020 (30%) subjects who reported a cough, we obtained sputum from 691 (68%) and identified 31 cases of active TB for a point prevalence of 917 (95% CI, 623–1302) per 100,000 prisoners. Evaluation of the two sputum smear samples failed to identify 74% of the TB cases, and 29% of the cases reported less than 2 weeks of symptoms. Obtaining a second culture identified an additional 7 (24%) cases. The prevalences of LTBI were 22.5% and 11.7% for male and female prisoners, respectively and duration of incarceration (in years) was associated with LTBI in male and female in the multivariable model (1.04, 95% CI, 1.01-1.07 and 1.34, 95% CI, 1.06-1.70, respectively). The prevalence of LTBI is 8.6% among newly incarcerated inmates, among whom LTBI prevalence significantly increased by 5% with each year of incarceration.

**Conclusions:**

Although the overall LTBI prevalence among inmates in Central-West Brazil is low, tuberculosis incidence is high (>1,800/100,00), likely due to the high force of infection among a largely susceptible inmate population. Efforts to reduce transmission in prisons may require mass screening for active TB, utilizing sputum culture in case-detection protocols.

**Electronic supplementary material:**

The online version of this article (doi:10.1186/s12879-015-0764-8) contains supplementary material, which is available to authorized users.

## Background

Worldwide, the prevalence of tuberculosis (TB) among prisoners is frequently an order of magnitude greater than that in the general population [1]. In Brazil, which has the world’s 4th largest prisoner population, the incidence of TB in prisons is approximately 20 times greater than that in the general population (>1,000 per 100,000 versus 46 per 100,000) [2,3]. Previous studies of Brazilian prisons have reported that the prevalence of active TB and latent TB infection (LTBI) range from 2 to 9% and from 40 to 73%, respectively [2-5]. The high incidence of TB in prisons contributes to a high rate of TB transmission among inmates, who serve as a persistent reservoir for spillover TB transmission into the general population [6,7].

International and national guidelines concerning TB control in prisons recommend systematic screening using standardized symptom assessment and chest radiography to identify individuals requiring further investigation [8-12]. In particular, there remain critical questions about: the yield of symptom screening, smear microscopy and culture; the number of sputum examinations that should be performed; and whether demographic attributes or reported risk factors can effectively identify high-risk individuals to target for screening for LTBI and active TB.

Few studies conducted in prisons in low- or middle-income countries have addressed the performances of smear and culture for screening active TB in the prisons [12-14]. In Mato Grosso do Sul state, no control measures other than passive case detection have been implemented, wherein symptomatic inmates are referred for chest radiography, smear and TB culture, all performed outside of the prisons.

Due to the high tuberculosis incidence, limited prison health budgets, and complex nature of conducting public health interventions in these settings, more evidence for mass screening for tuberculosis is needed. We implemented a large campaign in a network of 12 prisons in Central-West Brazil to assess the yield of the mass screening of inmates for active TB. Furthermore, we evaluated the prevalence of tuberculin skin test (TST) positivity in this population to identify risk factors and risk groups for TB infection and to facilitate future intervention strategies.

## Methods

### Study setting and design

**Figure 1:**
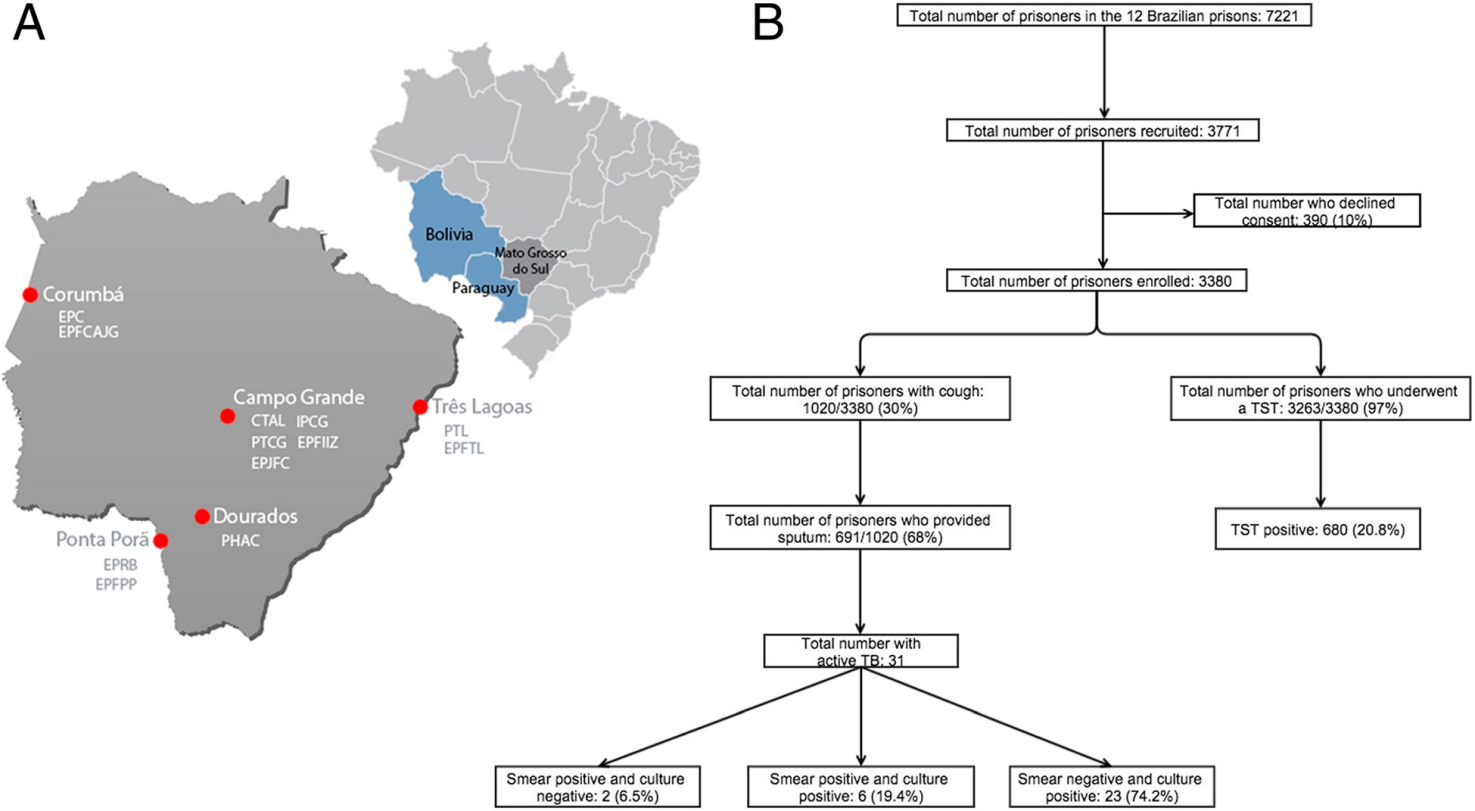
**Location of study prisons (A) and flow chart of the screening process for the detection of active and latent TB (B).** Abbreviations: EPC - Estabelecimento Penal de Corumbá, PTL - Penitenciária de Três Lagoas, EPRB - Estabelecimento Penal Ricardo Brandão, CTAL - Centro de Triagem Anízio Lima, PTCG - Presídio de Transito de Campo Grande, IPCG - Instituto Penal de Campo Grande, EPJFC - Estabelecimento Penal Jair Ferreira de Carvalho, PHAC - Penitenciária Harry Amorim Costa, EPFCAJG - Estabelecimento Penal Feminino Carlos Alberto Jonas Giordano, EPFTL - Estabelecimento Penal Feminino de Três Lagoas, EPFPP - Estabelecimento Penal Feminino de Ponta Porã, EPFIIZ - Estabelecimento Penal Feminino Irmã Irma Zorzi.

Mato Grosso do Sul is a state in Central-West Brazil that borders Paraguay and Bolivia. It is home to a population of 2.5 million people and has the highest rate of incarceration in the country, predominately due to drug-trafficking crimes. In 2013, there were 12,306 prisoners in the state, including 11,152 males and 1,154 females distributed between 37 penal institutions. In the “closed” subset of the system, in which prisoners do not leave the prison during their incarceration (in the contrast with the “open” system for lower-risk offenders), there were a total of 9,913 inmates at 22 penal institutions. The population of the closed system in the 5 largest cities in the state (Campo Grande, Corumbá, Dourados, Ponta Porã and Três Lagoas) was included in a cross-sectional study performed between January 2013 and December 2013 (Figure 1a). Twelve prisons were included in the study, with a total of 7,221 prisoners representing 73% of the prisoners in the closed system and 59% of the total prison population in the state. Of these 12 prisons, there were 8 male prisons (6,552 prisoners) and 4 female prisons (669 prisoners).

### Sample size calculation and study population

Prisoners who were 18 years of age and who consented to participate were included in the study. Screening for tuberculosis (reported here) was performed alongside parallel screening studies for HIV, Hepatitis B, Hepatitis C, and syphilis. The sample size was calculated based on the expected prevalence of HIV, assuming 2% for HIV with a variation of 1%, power of 80% and alpha-type error of 5%. The study population is 7,221 prisoners, and the sample size was 3,159 prisoners. We added 20% more individuals (total, 3,771 prisoners) to account for anticipated loss due to refusal to participate. Proportional stratified sampling was performed using each prison as a unit of randomization. On the data collection day, the prisoners were ordered numerically in ascending order from the lists provided by the prison and a list of random numbers was generated using the Epi-Info 6.04 software (Atlanta, GA, USA).

### Data collection

Data collection for all twelve prisons was carried out over a period of one year (01/07/2013 to 10/22/2013), with each prison being sequentially enrolled over a course of 1 to 3 weeks. Each participant underwent an interview utilizing a standardized questionnaire. The variables obtained during the interview included gender, marital status, education, smoking history, illicit drug use, diabetes, contact with a TB-positive individual (in the household or at other places), the presence of a Bacillus Calmette-Guérin (BCG) vaccine scar (as determined by inspecting the participant’s arm), previous incarceration, number of prisoners per cell, TB symptoms in the cell and time in prison. The participant’s race/skin color (i.e., white, black, indigenous, Asian or mixed) was self-reported.

### Tuberculin skin testing

Two tuberculin units (0.1 ml) of RT23 PPD (Staten Serum Institute, Copenhagen, Denmark) were injected intradermally into the volar aspect of the left forearm. After 48 hours, the maximum diameter of the palpable induration was measured by a trained TST reader. The TST was considered to be positive if the induration was ≥10 mm, except in HIV-positive patients, for whom an induration of ≥5 mm was considered to be positive.

### Smear and culture for *M. tuberculosis*

All patients reporting a cough were asked to provide sputum for an assessment of active TB. Two sputum samples were collected, including one spot sample after the interview and another the next morning. Smear microscopy and solid culture were utilized to test for *Mycobacterium tuberculosis* (*M. tuberculosis*). The smear was conducted according to the Ziehl-Neelsen technique and was read by a trained microscopist. After completing the smear, the samples were processed using the Swab method as described by Kudoh and Kudoh (1974) [15] and then decontaminated using the Petroff method, with a 5-minute exposure to 4% NaOH [16]. Culturing was performed using modified Ogawa medium (pH 6.4) [17], inspected daily for visible evidence of growth, and maintained for 60 days until it was considered negative Time to culture positivity was defined as the days from sputum collection to identification and confirmation of mycobacterial growth. Radiography was not available in the prisons, and a TB case was therefore defined as the presence of at least one positive smear or culture.

### Tuberculosis outcomes

To assess outcomes of tuberculosis cases identified during the screening study, we linked data on cases with cases reported in the National Notifiable Diseases Information System (SINAN). We classified outcomes according to World Health Organization definitions, as utilized by the Brazil national tuberculosis programme.

### HIV serology

All participants were offered HIV testing by serum ELISA, with positive tests confirmed by Western blot.

### Data analysis

All questionnaires were entered twice into Research Electronic Data Capture (REDCap), which is a secure online database. The questionnaires were compared to search for data entry errors. SAS version 9.2 (SAS Institute, Cary, NC, USA) and the R statistical software, version 3.1.1 (R Foundation for Statistical Computing, Vienna, Austria) were used to analyze the univariate and multivariate models. The prevalence of LTBI was expressed as the percentage among inmates screened, and the prevalence of active TB was expressed as the cases per 100,000 individuals, consistent with conventional metrics. We estimated the disease duration by dividing the prevalence to incidence of active TB. Tuberculosis incidence was estimated from notification data obtained from the National Notifiable Diseases Information System (SINAN), from which we identified all new cases of active TB reported in the 12 prisons during the study period. Dichotomized and categorical data were analyzed with the chi-squared test or Fisher’s exact test. For continuous variables, the t-test or analysis of variance (ANOVA) was utilized. Univariate analyses were performed to verify the associations between the dependent and independent variables. Poissson regression analysis was used to estimate the crude prevalence ratios (PRs). Those achieving a pre-specified level of significance (p < 0.05) were included in the multivariable analysis. Multi-level mixed Poisson regression models were used to estimate the risks of latent and active TB associated with sociodemographics and exposure variables for the individuals nested within the prisons.

### Ethical issues

All eligible participants provided written informed consent prior to study participation. The study was approved by the Research Ethics Committee at the Federal University of Grande Dourados (Number 191,877). Every active TB patient identified during the study was notified, underwent TB treatment and was provided with referrals. No preventive therapy was provided for LTBI because the Brazilian Ministry of Health does not recommend the treatment of LTBI in prisons [8].

## Results

Among the 3,771 prisoners recruited for the study, 391 (10%) refused to participate, and 3,380 were enrolled. Inmates refusing to participate had similar characteristics such as age, sex and reason for admission compared to those who screened. All enrolled participants completed the study protocol (Figure 1b). The mean age of the participants was 33.2 years (range, 18–80 years). The majority of these individuals was from the state of Mato Grosso do Sul (64%) and were men (85%). The prisoners’ self-reported racial groups included white (33%), mixed (51%), black (13%), indigenous (1%) and Asian (2%) (Table 1). There was considerable variation between prisons with respect to prior history of drug use during the previous year (17-70%) and TB (1-11%), inmates reporting knowing someone with TB (11-63%), previous incarceration (28 -75%) and mean duration of incarceration (4.4-29.9 months) (Additional file 1: Tables S1a and b). The most common reasons for the incarceration for men were drug trafficking (48%), theft (30%) and homicide (14%). Women were also most frequently incarcerated for drug trafficking (87%), theft (7%) and homicide (3%). Compared with women, men were more likely to have used drugs during the past year (54% versus 38%, p < 0.01), have a positive history of TB (6% versus 3%, p < 0.01), know someone with TB (43% versus 24%, p < 0.01), or have been previously incarcerated (62% versus 40%, p < 0.01), and their duration of incarceration was longer (20 months versus 12 months, p < 0.01) (Additional file 1: Tables S1a and b).

**Table 1:** **Sociodemographics, prison variables and tuberculin skin test results stratified by gender in Mato Grosso do Sul, Brazil (N = 3,380)**

	**Gender**		
	***(Number/percentage)***		
**Variables**	**Male**	**Female**	**P value**
	**N = 2,861**	**N = 519**	
**TST-positive**	620/2752 (22.5)	60/511 (11.7)	<0.01
**Active TB**	29/2861 (1)	2/519 (0)	0.2
**Reason for admission**			<0.01
Drug trafficking	1142/2360 (48)	383/437 (88)	
Theft	741/2360 (31)	30/437 (7)	
Homicide	324/2360 (14)	14/437 (3)	
Sexual abuse	64/2360 (3)	1/437 (0)	
Other	87/2360 (4)	9/437 (2)	
**Sociodemographics**			
**Age, years, mean ± SD**	32 ± 10	32 ± 10	0.52
**Marital status, single**	1522/2840 (54)	332/506 (66)	<0.01
**Race**			<0.01
White	912/2747 (33)	137/470 (29)	
Mixed	1366/2747 (50)	283/470 (60)	
Black	370/2747 (14)	34/470 (7)	
Indigenous	37/2747 (1)	4/470 (1)	
Asian	62/2747 (2)	12/470 (3)	
**Resides in MS**	1889/2861(66)	277/519 (53)	<0.01
**Less than 4 years of schooling**	1195/2794 (43)	282/514 (55)	<0.01
**Diabetes**	78/2399 (3)	22/494 (5)	0.18
**Current smoker**	1551/2830 (55)	284/519 (55)	0.97
**Drug use over the last year**	1543/2861(54)	199/519 (38)	<0.01
**Previous TB**	176/2817 (6)	14/518 (3)	<0.01
**HIV-positive**	45/2847 (1.6)	10/518 (1.9)	0.56
**Prison**			
**Previously incarcerated**	1758/2836 (62)	207/517 (40)	<0.01
**Knows someone with TB**	1196/2784 (43)	124/511 (24)	<0.01
**Prisoners per cell, mean ± SD**	16 ± 12	22 ± 15	<0.01
**Duration of incarceration, months, mean ± SD**	20 ± 27	12 ± 13	<0.01
**Other prisoners coughing in the cell**	845/2830 (30)	211/517 (41)	<0.01

Of the 3,380 study participants, 1,020 reported a cough of any duration, and 691 (68%) were able to produce sputum. Thirty-one participants had bacteriologically confirmed TB, corresponding to a point prevalence of 917 (95% CI, 623–1302) per 100,000 prisoners. Among the 31 cases, 29 (93%) were culture-positive, and two were positive by smear only. Six cases were positive by both culture and smear, and twenty-three of the 31 cases (74%) were culture-positive and smear-negative (Figure 1b). The median time to culture positivity was 34 days (range, 18–66 days). Among the smear-positive cases, 75% were positive according to the first smear, and a second smear identified the remaining 25%. For the culture-positive cases, 76% of the first specimens tested positive, with an additional 24% positive by the second culture only.

Among the TB patients, 29 (96.2%) were men (median age, 32 years; range, 22–55 years), and 16 (51.6%) were from a single prison (EPJFC) (Additional file 1: Tables S1a and b). Of the 27 patients notified and identified in the SINAN electronic database, 21 (78%) completed the treatment and were cured, 2 (7%) died, 2 (7%) defaulted, and 2 (7%) was transferred. Among the 31 patients, 1 patient had already been diagnosed with TB 6 months ago and four reported having had prior TB (48 months, 36 months, 24 months and 12 months ago). In 2013, 142 TB cases from 12 prisons were reported to SINAN, which represented an incidence of 1,839 per 100,000 inhabitants. Comparing TB notifications for 2013 with the point prevalence estimated here, the estimated duration of TB prior to diagnosis was 6 months.

**Figure 2:**
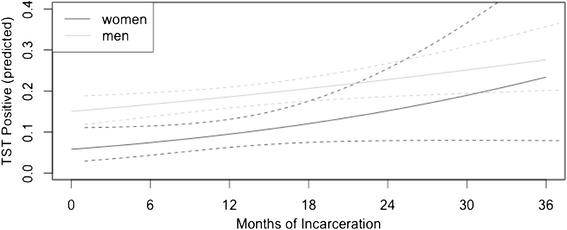
**Three-year predicted prevalence of TST-positive results for the prisoners with no past history of incarceration.**

The prevalence of TST positivity among the different prisons ranged from 3.0% (EPFTL prison) to 32.0% (EPJFC prison) and was higher among men (22.5%) than women (11.7%) (p < 0.01). In a subset of 144 prisoners with ≤1 month of current incarceration and no prior history of imprisonment, the prevalence of TST positivity was 7.6%. Its prevalence increased significantly by 5% per year with time spent in prison for the entire prisoner population, with a greater slope of increase for women than for men (Figure 2).

Among the male prisoners, TST positivity was independently associated with increasing age (adjusted prevalence ratio [APR], 1.02, 95% confidence interval [CI], 1.01-1.02), race (with white race as the reference; mixed: APR, 1.34, 95% CI, 1.10-1.63; black: APR, 1.43, 95% CI, 1.10-1.86; Asian: APR, 1.80, 95% CI, 1.16-2.90; indigenous: APR, 1.25, 95% CI, 0.85-1.85), drug use within the past year (APR, 1.29, 95% CI, 1.08-1.54), and years of incarceration (APR, 1.04, 95% CI, 1.01-1.07) (Table 2). Among the female prisoners, TST positivity was independently associated with increasing age (APR, 1.03, 95% CI, 1.00-1.06), previous TB diagnosis (APR, 3.79, 95% CI, 1.52-9.48), previous incarceration (APR, 2.05, 95% CI, 1.16-5.96), number of prisoners per cell (APR, 1.02, 95% CI, 1.01-1.04), knowing someone with TB (APR, 2.05, 95% CI, 1.16-3.52) and years of incarceration, among those without incarceration history (APR, 1.34, 95% CI, 1.06-1.70) (Table 2).

**Table 2:** **Risk factors associated with LTBI among male and female prisoners**

	**Male (N = 2752)**		**Female (N = 511)**	
**Variables**	**Crude PR**	**Adjusted PR**	**Crude PR**	**Adjusted PR**
**Sociodemographics**				
**Age, per year**	0.99 (0.98-0.99)	1.02 (0.67-1.53)	1.03 (1.00-1.05)	1.03 (1.01-1.06)
**Marital status, single**	0.89 (0.76-1.04)		0.84 (0.48-1.48)	
**Race**				
White				
Mixed	1.31 (1.08-1.59)	1.34 (1.10-1.63)	1.36 (0.69-2.71)	
Black	1.40 (1.08-1.81)	1.43 (1.10-1.86)	2.32 (0.89-6.04)	
Indigenous	1.28 (0.63-2.60)	1.25 (0.59-2.68)		
Asian	1.72 (1.07-2.76)	1.80(1.12-2.90)		
**Resides in MS**	0.83 (0.71-0.98)		1.13 (0.67-1.89)	
**No education**	0.82 (0.69-0.96)		0.68 (0.41-1.13)	
**Diabetes**	1.14 (0.71-1.82)		0.34 (0.05-2.41)	
**Current smoker**	1.21 (1.03-1.42)		1.74 (1.01-2.98)	
**Drug use over the last year**	1.21 (1.02-1.42)	1.29 (1.08-1.54)	1.05 (0.62-1.75)	
**BCG scar**				
**Previous TB**	1.50 (1.14-1.98)		3.14 (1.42-6.91)	3.79 (1.52-9.47)
**HIV-positive**	1.34 (0.79-2.27)		0.90 (0.13-6.44)	
**Prison**				
**Previously incarcerated**	1.24 (1.05-1.48)		1.39 (0.83-2.33)	2.44 (1.17-5.10)*
**Knows someone with TB**	1.16 (0.98-1.37)		2.55 (1.54-4.24)	2.02 (1.16-3.52)
**Prisoners per cell, per person**	0.99 (0.98-1.00)		1.02 (1.01-1.04)	1.02 (1.01-1.04)
**Duration of incarceration, years**	1.04 (1.01-1.07)	1.04 (1.01-1.07)	1.06 (0.87-1.31)	1.34 (1.06-1.70)*
**Other prisoners coughing in the cell**	1.04 (0.88-1.24)		1.05 (0.61-1.78)	

*Adjusted for significant interaction term between these variables (p = 0.01).

## Discussion

Previous studies in Brazilian prisons have found prevalences of LTBI ranging from 49% to 73% [2-4,18]. This multicenter study, which was conducted in 12 prisons with 3,360 inmates of both genders in Mato Grosso do Sul, revealed a comparatively low prevalence of LTBI that varied from 15-33% in the male prisons and from 3-16% in the female prisons. In particular, during the first month of incarceration, the prevalence was very low (7.9% in males and 8.3% in females), which indicates that a large number of individuals who enter prison are susceptible to TB infection. The low prevalence of LTBI combined with the high point prevalence and incidence (951 and 1839 per 100,000, respectively) highlight the urgent need for new interventions in these settings to reduce TB transmission.

In this study, previous incarceration was a significant independent risk factor for LTBI among women, and trended towards significance as a risk factor for men. A number of studies in prisons have demonstrated that previous incarceration [19,20] is associated with TST positivity. In São Paulo, 55.1% of detainees have been identified as infected individuals with previous arrests, and 75.6% have LTBI [21]. Previous incarceration has been strongly associated with TB among the urban population, and genotypic data indicate that there is a considerable spillover into the Dourados general urban population [22]. Moreover, duration of incarceration was associated with TST positivity, controlling for other demographic risk factors, which further implicates prisons as the source of tuberculosis infection rather than high-risk characteristics of the inmate population.

Taken together, the low prevalence of TB among individuals who were new to the prison system and the association between the duration of incarceration and LTBI positivity imply that prisons are critical to TB transmission. This finding underscores the need to implement measures to control TB in this setting. This goal may require a combination of biomedical interventions (improving diagnostic capacity, implementing active case detection strategies) and structural interventions (reducing crowding, improving ventilation and prison conditions) to achieve a substantial impact on the extraordinary burden of TB in Brazilian prisons [23].

We found a lower prevalence of TST-positive inmates in the Centro de Triagem (CTAL = 16%) and Presidio de Trânsito (PTCG = 15%) prisons, where prisoners are typically held temporarily; these facilities generally serve as entrances into the prison system. The highest prevalence rates were observed in the prisons in which the inmates remained for longer periods of time, such as the Estabelecimento de Segurança Máxima prison (EPJFC = 32%); these prisons also had higher TB notification rates (Additional file 1: Tables S1a and b). In these initial, more temporary prisons, TB screening may be instituted using smear and culture in symptomatic inmates, and an initial TST should be conducted in asymptomatic individuals, who should potentially be monitored during incarceration.

Currently, the Brazilian Ministry of Health does not recommend treating LTBI in inmates with a positive TST [8]. Our findings of the low LTBI prevalence upon prison entry and high risk thereafter suggest that individuals may be screened upon incarceration and periodically thereafter to detect the development of infections. Recent infection, rather than late reactivation, likely drives the majority of tuberculosis in this setting. Thus, periodic screening, either annually or among cellmates of a detected case, combined with isoniazid preventive therapy may be an effective intervention.

In the prisons examined in this study, as is common throughout Brazil, TB diagnoses are made when inmates present to the prison clinic with relevant symptoms (i.e., passive case finding). Actively searching for TB cases may prove effective, as demonstrated by this study, which detected 31 cases of active TB among 3,380 prisoners screened. We observed that the two smears failed to detect 74% of cases. This observation may indicate that individuals were diagnosed earlier in their course of illness, but it also suggests that a smear is insufficient for active case detection in this setting. Additionally, because a quarter of the cases were only diagnosed following a second culture, we advocate the collection of at least two specimens when performing mass screening in prisons. Further studies are needed to evaluate various diagnostic strategies, the yields of various intervals of screening, and their cost-effectiveness.

The prevalence of LTBI in our study was lower than that identified in other studies [2,18]. This difference is likely due to the low incidence of TB in the general population of the state of Mato Grosso do Sul. Only two studies have estimated the prevalence of TST in two prisons located in the first and third most populous cities in Brazil (São Paulo and Salvador, respectively), in which the majority of prisoners are former residents of slums, which are areas with high LTBI rates.

The results of this study should be interpreted within the context of the limitations of the data. We screened all individuals who reported cough of any duration, which while a more liberal screening definition than the WHO symptom criteria (cough > 2 weeks), may nevertheless fail to identify a substantial proportion of tuberculosis cases in this population [12,14]. Additionally, one-third of the patients reporting a cough were unable to produce sputum, and resources for sputum induction in the prisons were not available. Radiography is not available in prisons, and we were unable to assess the utility of this important diagnostic tool. We used solid (Ogawa) media for culture, due to low costs and lower laboratory safety risks; this procedure may be less sensitive than liquid media culture, but is more widely available in resource-limited settings and therefore more generalizable. For all of these reasons, our estimate of tuberculosis prevalence is likely low. The majority of cases were detected in just one prison, and it is unclear whether this was a higher-burden prison or whether these were point-source outbreaks. Genotyping of isolates may further clarify this issue.

## Conclusions

The combination of the high TB prevalence identified in the prisons evaluated, which is similar to reports from other prisons in Brazil and in other countries [1-5], with the large proportion of susceptible individuals initially entering into the prison system contribute to a high force of infection in this setting. Recent transmission, rather than reactivation, likely is driving the tuberculosis epidemic in these prisons, and interventions will need to be focused towards interrupting ongoing transmission. This situation represents a social justice crisis, and mass screening for active and latent TB infections must be implemented in Brazilian prisons.

## Additional file

Additional file 1: Table S1a. Characteristics of 8 male Brazilian prisons (N=2,861). **Table S1b.** Characteristics of 4 female Brazilian prisons (N=519).
