# Supplementary material for: Active and latent tuberculosis in Brazilian correctional facilities: a cross-sectional study
Source: BMC Infect Dis. 2015 Jan 22;15:24. doi: 10.1186/s12879-015-0764-8 (PMC4307675; doi:10.1186/s12879-015-0764-8)
Supplement: Additional file 1: Table S1a. — Characteristics of 8 male Brazilian prisons (N=2,861). Table S1b. Characteristics of 4 female Brazilian prisons (N=519). [file 12879_2015_764_MOESM1_ESM.docx]

**Additional file 1**

| **Table S1a – Characteristics of 8 male Brazilian prisons (N=2,861)** | | | | | | | | | | | | | |
| --- | --- | --- | --- | --- | --- | --- | --- | --- | --- | --- | --- | --- | --- |
| **Variables** | **Prison**  ***(Number/percentage)*** | | | | | | | |  | |  | |  |
|  | **EPC**  **N=263** | **PTL**  **N=283** | **EPRB**  **N=252** | **CTAL**  **N=116** | **PTCG**  **N=286** | **IPCG**  **N=518** | **EPJFC**  **N=604** | **PHAC**  **N=539** | |  | | **P value** |  |
| **Positive TST** | 86/261 (33) | 51/277 (18) | 68/249 (27) | 18/116 (16) | 60/276 (22) | 71/485 (15) | 180/568 (32) | 86/520 (17) | |  | | <0.01 |  |
| **Active TB** | 4/263 (2) | 3/283 (1) | 1/252 (0) | 0/116 (0) | 1/286 (0) | 3/518 (1) | 16/604 (3) | 1 (0) | |  | | <0.01 |  |
| **Sociodemographics** |  |  |  |  |  |  |  |  | |  | |  |  |
| **Age, years, mean±SD** | 32.6±10 | 31.8±10 | 33.8±10.6 | 35.9±10.1 | 28.9±9.6 | 33.9±10 | 30.8±8 | 31.5±9.3 | |  | | <0.01 |  |
| **Marital status, single** | 116/262 (44) | 138/282 (49) | 130/251 (52) | 64/115 (56) | 117/283 (41) | 260/512 (51) | 241/597 (40) | 252/538 (47) | |  | | <0.01 |  |
| **Race**  White  Mixed  Black  Indigenous  Asian | 74/260 (28)  138/260 (53)  33/260 (13)  8/260 (3)  7/260 (3) | 71/219 (25)  120/219 (42)  22/219 (8)  1/219 (0)  5/219 (2) | 94/252 (37)  136/252 (54)  19/252 (8)  2/252 (1)  1/252 (0) | 41/116 (35)  64/116 (55)  9 /116 (8)  0 /116 (0)  2 /116 (2) | 76/286 (27)  145/286 (51)  49/286 (17)  6/286 (2)  9/286 (3) | 139/514 (27)  275/514 (53)  78/514 (15)  7/514 (1)  13/514 (3) | 178/561 (29)  262/561 (43)  105/561 (17)  1/561 (0)  15/561 (2) | 239/539 (44)  225/539 (42)  53/539 (10)  12/539 (2)  10/539 (2) | |  | | <0.01 |  |
| **Resides in MS** | 180/263 (68) | 147/283 (52) | 119/252 (47) | 85/116 (73) | 206/286 (72) | 365/518 (71) | 415/604 (69) | 372/539 (69) | |  | | <0.01 |  |
| **Less than 4 years of**  **Schooling** | 111/255 (44) | 124/277 (45) | 144/248 (58) | 59/111 (47) | 134/278 (48) | 190/505 (38) | 241/586 (41) | 199/534 (37) | |  | | <0.01 |  |
| **Diabetes** | 6/243 (3) | 11/232 (5) | 7/207 (3) | 6/111 (5) | 6/248 (2) | 21/467 (5) | 10/452 (2) | 11/439 (3) | |  | | 0.26 |  |
| **Current smoker** | 156/262 (60) | 147/281 (52) | 110/251 (44) | 48/101 (48) | 171/285 (60) | 254/516 (49) | 365/598 (61) | 300/536 (56) | |  | | <0.01 |  |
| **Drug use over the last**  **Year** | 110/263 (42) | 160/283 (57) | 93/252 (37) | 47/116 (41) | 158/286 (55) | 272/518 (53) | 420/604 (70) | 283/539 (53) | |  | | <0.01 |  |
| **Previous TB** | 12/262 (5) | 13/283 (5) | 7/51 (3) | 6/89 (7) | 5/273 (2) | 39/518 (8) | 64/602 (11) | 30/539 (6) | |  | | <0.01 |  |
| **HIV-positive** | 5/261 (2) | 5/282 (2) | 3/250 (1) | 3/116 (3) | 5/286 (2) | 8/516 (2) | 6/601 (1) | 10/535 (2) | |  | |  |  |
| **Prison** |  |  |  |  |  |  |  |  | |  | |  |  |
| **Previously incarcerated** | 156/262 (60) | 169/283 (60) | 98/252 (39) | 71/102 (70) | 132/281 (47) | 333/517 (64) | 450/602 (75) | 349/537 (65) | |  | | <0.01 |  |
| **Knows someone with TB** | 94/258 (36) | 90/281 (32) | 75/250 (30) | 58/92 (63) | 29/269 (11) | 267/511 (52) | 346/592 (58) | 237/531 (45) | |  | | <0.01 |  |
| **Prisoners per cell,**  **mean ±SD** | 17.1±9.4 | 5.7±2.1 | 15.2±5.3 | 9.2±2.3 | 14.4±9.0 | 35.4±13.5 | 11.3±4.8 | 9.7±4.1 | |  | | <0.01 |  |
| **Duration of incarceration,**  **months, mean±SD** | 15.8±14.1 | 21.2± 25.7 | 12.3±13.3 | 14.5±21.6 | 4.4±5.1 | 23.1±30.2 | 29.9±36.6 | 20.8± 24.1 | |  | | <0.01 |  |
| **Other prisoners coughing in the cell** | 49/259 (19) | 32/283 (11) | 92/252 (37) | 12/115 (10) | 95/273 (35) | 201/515 (39) | 254/596 (43) | 110/537 (21) | |  | | <0.01 |  |

Abbreviations: EPC - Estabelecimento Penal de Corumbá, PTL - Penitenciária de Três Lagoas, EPRB - Estabelecimento Penal Ricardo Brandão, CTAL - Centro de Triagem Anízio Lima, PTCG - Presídio de Transito de Campo Grande, IPCG - Instituto Penal de Campo Grande, EPJFC - Estabelecimento Penal Jair Ferreira de Carvalho, PHAC - Penitenciária Harry Amorim Costa, MS - Mato Grosso do Sul, TST - tuberculin skin test

| **Table S1b - Characteristics of 4 female Brazilian prisons (N=519)** | | | | | | |
| --- | --- | --- | --- | --- | --- | --- |
| **Variables** | **Prison**  ***(Number/percentage)*** | | | | | |
|  | **EPFCAJG**  **N=81** | **EPFTL**  **N=76** | **EPFPP**  **N=94** | **EPFIIZ**  **N=268** |  | **P value** |
| **Positive TST** | 8 /81(10) | 2/75 (3) | 7/91(8) | 43/264 (16) |  | 0.02 |
| **Active TB** | 0 (0) | 0 (0) | 0 (0) | 2 (1) |  | 0.60 |
| **Sociodemographics** |  |  |  |  |  |  |
| **Age, years, mean±SD** | 31.9±9.8 | 31.2±10.2 | 32±11.9 | 31.5±9.3 |  | <0.01 |
| **Marital status, single** | 31/81 (38) | 28/76 (37) | 32/94 (34) | 83/255 (33) |  | 0.77 |
| **Race**  White  Mixed  Black  Indigenous  Asian | 26/80 (33)  49/80 (61)  4/80 (5)  1/80 (1)  0/80 (0) | 28/76 (37)  43/76 (56)  2/76 (3)  1/76 (1)  2/76 (3) | 27/93 (29)  51/93 (55)  12/93 (13)  1/93 (1)  2/93 (2) | 56/221 (25)  140/221 (63)  16/221 (7)  1/221 (1)  8/221 (4) |  | 0.26 |
| **Resides in MS** | 30/81 (37) | 42/76 (55) | 39/94 (42) | 166/268 (62) |  | <0.01 |
| **Less than 4 years of**  **schooling** | 49/81 (61) | 33/76 (43) | 59/92 (64) | 141/265 (53) |  | 0.04 |
| **Diabetes** | 5/77 (7) | 1/70 (1) | 1/94 (1) | 15/253 (6) |  | 0.11 |
| **Current smoker** | 39/81 (48) | 42/76 (55) | 49/94 (52) | 154/268 (58) |  | 0.48 |
| **Drug use over the last year** | 14/81 (17) | 27/76 (36) | 39/94 (42) | 119/268 (44) |  | <0.01 |
| **Previous TB** | 1/81 (1) | 1/76 (1) | 2/94 (2) | 10/267 (4) |  | 0.49 |
| **HIV-positive** | 2/81 (3) | 1/76 (1) | 1/93 (1) | 6/268 (2) |  | 0.86 |
| **Prison** |  |  |  |  |  |  |
| **Previously incarcerated** | 22/81 (27) | 28/76 (37) | 28/92 (30) | 129/268 (48) |  | <0.01 |
| **Knows someone with TB** | 16/80 (20) | 12/75 (16) | 24/94 (26) | 72/262 (28) |  | 0.16 |
| **Prisoners per cell,**  **mean ±SD** | 4±1 | 6.5±2.1 | 10.3±2.7 | 35.1±8.5 |  | <0.01 |
| **Duration of incarceration,**  **months, mean±SD** | 13.7±9.8 | 6.7± 6.3 | 10.9±10.4 | 13.9±14.9 |  | <0.01 |
| **Other prisoners coughing in**  **the cell** | 7/81 (9) | 20/76 (26) | 38/94 (40) | 146/266 (55) |  | <0.01 |

Abbreviations: EPFCAJG - Estabelecimento Penal Feminino Carlos Alberto Jonas Giordano, EPFTL - Estabelecimento Penal Feminino de Três Lagoas, EPFPP - Estabelecimento Penal Feminino de Ponta Porã, EPFIIZ - Estabelecimento Penal Feminino Irmã Irma Zorzi, MS - Mato Grosso do Sul, TST - tuberculin skin test.
